# *Krüppel*-like Factors in the Gastrointestinal Tract

**DOI:** 10.3390/cells14191513

**Published:** 2025-09-28

**Authors:** Dharmendra Bhargava, Anchal Neha Bhargava, Jonathan P. Katz

**Affiliations:** Division of Gastroenterology, University of Pennsylvania Perelman School of Medicine, Philadelphia, PA 19104, USA; bhargavd@pennmedicine.upenn.edu (D.B.); anchalnehabhargava@gmail.com (A.N.B.)

**Keywords:** *Krüppel*-like factors (KLFs), gastrointestinal tract, transcriptional regulator

## Abstract

**Highlights:**

**What are the main findings?**

- The Krüppel-like factors (KLFs) occupy nodal positions at the intersection of transcriptional regulation, metabolism, stress response, and cell fate determination in both normal gastrointestinal (GI) physiology and disease
- The functions of the KLFs within the GI tract are highly context-dependent.

**What is the implication of the main finding?**

- Understanding specific KLF functions in the GI tract is essential.
- The KLFs can potentially be targeted for GI diseases.

**Abstract:**

The *Krüppel*-like factors (KLFs) are a family of transcriptional regulators that play crucial roles in regulating diverse cellular processes including development, proliferation, differentiation, metabolism, and carcinogenesis across various tissues. KLFs play pivotal roles in gastrointestinal pathologies, and exhibit tissue- and cell-type-specific expression patterns throughout the gastrointestinal tract. During gastrointestinal (GI) development, KLFs orchestrate the transition from embryonic to adult gene programming, with specific family members being essential for proper organogenesis and tissue formation. KLFs also function as context-dependent modulators of GI homeostasis, inflammation, and carcinogenesis in adult tissues and interact with major signaling pathways such as PI3K/AKT, NF-κB, Wnt, Notch, MAPK, and TGF-β. This review comprehensively examines the roles of KLFs in GI health and disease, focusing on their expression patterns, regulatory mechanisms, function in normal homeostasis, and therapeutic implications for gastrointestinal disorders.

## 1. Introduction

**Figure 1:**
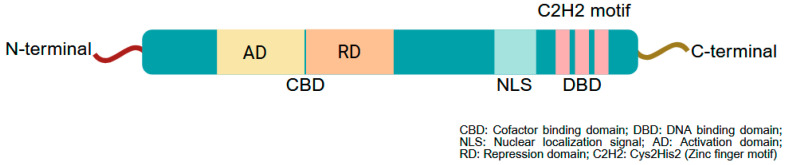
Typical structure of KLFs. KLFs possess a typical nuclear receptor structure, characterized by a cofactor binding site (CBD), a nuclear localization signal (NLS), and a highly conserved DNA-binding domain (DBD). The DBD contains two zinc fingers with 25 amino acids each and a third zinc finger with 23 amino acids. These zinc fingers recognize three unique base pairs in the DNA sequence, typically following the general form NCR CRC CCN (where N is any base and R is a purine). The CBD functions as either an activating or repressive domain depending on the cell type, cellular environment, and context. Created with BioRender.com.

The *Krüppel*-like factors (KLFs) are a family of transcriptional regulators, linked by their triple zinc-finger DNA binding domains [1,2]. A typical domain schematic diagram is presented in Figure 1. KLF family members are expressed broadly in a number of tissues, including the gastrointestinal tract, where they regulate a vast array of cellular processes such as development, cell proliferation and differentiation, metabolism, and carcinogenesis [3,4]; some of these KLFs are expressed ubiquitously while others are tissue restricted or localized further to specific cell types within a tissue. All KLFs except for KLF8, which is located on the X chromosome, are found on autosomes, with their chromosomal distribution shown in Table 1. Also, based on interacting cofactors and gene modulatory activity, the mammalian KLFs have been divided into three groups [2]: Group 1 members (KLF 3, 8, and 12) are typically repressors that interact with CtBP1 and CtBP2; Group 2 members (KLFs 1, 2, 4, 5, 6, and 7) are generally activators; and Group 3 members (KLFs 9, 10, 11, 12, 13, and 16) share the common co-repressor Sin3A and histone deacetylase complex [5,6]. KLFs 15 and 17 do not have defined protein interaction motifs [7].

Nonetheless, these classifications are not ironclad, and many KLFs can activate or repress gene transcription depending on context, including the presence of specific co-factors [8,9]. In recent years, KLFs have emerged as key regulators of gastrointestinal biology and potential therapeutic targets for gastrointestinal disorders.

## 2. Expression of KLFs in Gastrointestinal Tract

KLFs are expressed in various tissues, including in the gastrointestinal tract, where they serve as crucial regulators of gastrointestinal homeostasis, health, and function [1,2,10]. In turn, the expression of KLFs in the gastrointestinal tract is influenced by factors such as protein kinases, growth factors, circadian clock proteins, interleukins, nuclear receptors, and microRNAs. These factors modulate KLF’s transcriptional activity and stability by affecting promoter binding, post-translational modifications, and interactions with other proteins [11]. KLFs may be expressed broadly or may be tissue- and cell-type specific.

Among the KLFs, KLF4 (previously gut-enriched *Krüppel*-like factor or GKLF) and KLF5 (previously intestinal-enriched *Krüppel*-like factor or IKLF) are the most extensively studied in the context of homeostasis, physiology, and disease in the luminal gastrointestinal (GI) tract and pancreas [12,13,14]. Within GI epithelia, KLF4 is typically restricted to differentiating or differentiated cells, including committed basal and suprabasal cells of the squamous-lined esophagus [15,16,17], the middle to upper portions of the gastric units [18,19,20], small intestinal villi, and the upper regions of the colonic crypts [21,22,23], where KLF4 typically functions to drive cellular differentiation. Nonetheless, the expression of the KLFs is dynamic and stimulus responsive; for example, KLF4 expression is induced by inflammatory cytokines such as TNF-α and IL-6, which are involved in intestinal inflammation and immunity. Additionally, circadian clock proteins like BMAL1 and REV-ERBα regulate KLF4 expression, controlling the diurnal rhythm of intestinal physiology and metabolism [24]. In contrast, KLF5 is expressed in the basal layer of the squamous epithelium in the esophagus and in proliferating crypt cells of the small intestine and colon [25,26,27]. KLF5 plays a critical role in maintaining intestinal stem cells and promoting intestinal epithelial cell proliferation [25,26,27]. However, excessive expression of both KLF5 and KLF4 can lead to the development of intestinal tumors and esophageal squamous cell carcinoma [3].

Other KLFs are also expressed in the gastrointestinal tract, though their roles are less well defined. KLF6, including through its different splice variants, has been liked to liver pathophysiology [28,29,30]. KLF6 is also expressed in both epithelial and stromal cells in the gastrointestinal tract, and its overexpression has been associated with the development of intestinal tumors [11,31,32]. In the pancreas, both KLF4 and KLF5 promote acinar-to-ductal reprogramming in response to injury [13,14,33]. KLF2 and KLF10 are expressed in immune cells in the gastrointestinal tract and are essential for their normal function [34,35,36], while KLF9 is expressed in intestinal epithelia, where it plays an important for cell renewal and lineage determination [37]. KLF15 is expressed in both small intestine and liver and controls bile acid synthesis [38]. Interestingly, in *C. elegans* there are only three KLFs, rather than the 17 in mammals, and *klf-1* and *klf-3* are highly expressed in intestine where they regulate fat accumulation as well as lifespan in response to dietary restriction [39,40,41].

## 3. KLFs in Gastrointestinal Inflammation and Injury

Inflammation, injury, and eventual healing are complex, multistep processes involving immune cells, blood vessels, epithelial cells, and molecular mediators that facilitate crosstalk and guide tissue repair to restore structure and function [42,43]. Both acute and chronic inflammation are relevant for gastrointestinal diseases [44]. While acute inflammation is a rapid, localized response often triggered by infections, injuries, or allergens that aim to limit tissue damage and initiate the healing process in order to restore normal function, chronic inflammation is a persistent, systemic response that can last for months or even years, often continuing after the initial trigger is gone. The process of acute wound healing occurs in three distinct phases: inflammatory, proliferative, and remodeling [45,46]. The inflammatory phase, lasting a few days, begins with the release of mediators such as histamine and cytokines. These mediators cause blood vessel dilation and increase blood flow to the wound. Neutrophils, the first responders, clear debris and pathogens through phagocytosis and secrete chemokines and interleukins to recruit additional immune cells [47].

**Figure 2:**
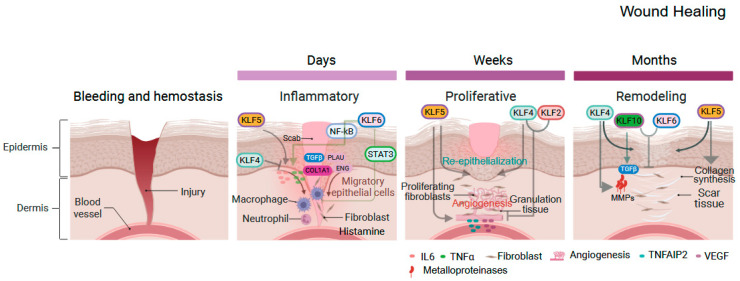
Roles of KLFs in gastrointestinal inflammation and healing. The repair process proceeds through three phases: inflammatory, proliferative, and remodeling. In the inflammatory phase, typically lasting a few days, KLF5 and KLF6 drive cytokine recruitment via IL-6, TNFα, and NF-κB, while KLF4 reduces inflammation and KLF6 shows dual effects through NF-κB or STAT3. In the proliferative phase (days to weeks), fibroblasts synthesize collagen and promote angiogenesis; KLF5 enhances angiogenesis via VEGF, whereas KLF4 and KLF2 inhibit it. KLF4 promotes fibroblast migration and collagen deposition, and KLF5 facilitates epithelial proliferation and migration during re-epithelialization. In the remodeling phase (weeks to months), tissue strength is restored by ECM remodeling and collagen realignment. KLF10 and KLF6 suppress scar formation by limiting fibroblast proliferation and collagen synthesis, while KLF4 and KLF5 drive ECM remodeling through MMP induction and collagen turnover. Created with BioRender.com.

KLFs modulate gastrointestinal inflammation, acting as either pro- or anti-inflammatory regulators depending on context, thereby maintaining a balance that facilitates progression to subsequent healing stages (Figure 2). KLF2 modulates the actions of monocytes, T-lymphocytes, and vascular endothelial cells [48,49] and promotes neutrophil activation via HIF1, producing pro-inflammatory cytokines and ROS [50], while also conferring anti-inflammatory properties to endothelial cells by inducing eNOS and thrombomodulin in presence of TNFα [51,52]. KLF4 exhibits context-dependent roles including via the NF-κB pathway. In macrophages stimulated with IFN-γ and LPS, KLF4 binds to the NF-κB p65 subunit, activating the NOS2 gene and promoting pro-inflammatory responses, while in epithelial cells, KLF4 triggers the production of pro-inflammatory cytokines, contributing to acute colitis in dextran sulfate sodium-treated mice and to esophageal squamous cell cancer [53,54,55]. However, in endothelial cells, KLF4 reduces inflammation by enhancing anti-inflammatory and anti-thrombotic factors, protecting cells from damage, and the absence of KLF4 amplifies TNFα-induced inflammation through increased vascular cell adhesion molecule 1 (VCAM1) expression [56]. Wound healing triggers the expression of KLF5 and KLF6, which in turn regulate pro-inflammatory mediators, such as IL-6 and TNF-alpha, across various cell types and contexts [57]. These factors also modulate the expression of genes involved in the cellular injury response and tissue repair, including TGFβ1, COL1A1, PLAU, and ENG, particularly during the early stages of wound healing [28]. Interestingly, KLF6 regulates both pro-inflammatory and anti-inflammatory signaling in intestinal myeloid cells through distinct signaling pathways and can thus drive inflammation by simultaneously activating and repressing these pathways. Acting via NF-kB, KLF6 promotes macrophage activation via expression of pro-inflammatory genes, while KLF6 also negatively regulates STAT3 transcriptional activity to repress IL-10-induced *Socs3* expression in myeloid cells [31].

Fibroblasts are key players in wound healing, migrating to the injured site to synthesize collagen, which provides a scaffold and structural strength to the repaired tissue [42,43]. In parallel, blood vessels begin to grow, delivering oxygen and nutrients to the healing area. KLF2 and KLF4 inhibit angiogenesis by suppressing the expression of pro-angiogenic factors [58], Adding to this complexity, KLF4 inhibits angiogenesis by elevating miR-15a levels in endothelial cells and vascular smooth muscle cells [59]. Similarly, sustained KLF4 overexpression in endothelial cells impairs angiogenic sprouting by modulating Notch signaling, further supporting an anti-angiogenic role for KLF4 [60]. In contrast, KLF5 promotes angiogenesis by upregulating vascular endothelial growth factor (VEGF) [61,62] or partially through TNF-α-induced protein 2 (TNFAIP2) [63]. As the wound heals, granulation tissue—comprising fibroblasts, new blood vessels, and extracellular matrix—fills the wound bed. KLFs regulate the proliferation and migration of various cell types involved in wound healing, such as fibroblasts and keratinocytes. Notably, KLF4 has been shown to enhance fibroblast migration and collagen production [64], thereby facilitating the formation of granulation tissue.

Both KLF4 and KLF5 contribute to re-epithelialization, the process of forming a new epithelial layer over the wound bed, by stimulating epithelial cell migration, proliferation, and differentiation [65]. Interestingly, proliferative epithelial cells exhibit higher levels of KLF5, whereas differentiating cells predominantly express KLF4 [66]. The remodeling phase, the longest phase of wound healing, can last for months, strengthening and reorganizing the newly formed tissue [67]. KLFs are integral to scar formation, as they regulate the expression of genes involved in extracellular matrix (ECM) synthesis and remodeling, including collagen, fibronectin, and matrix metalloproteinases (MMPs) [68]. For example, KLF4 modulates the expression of MMPs, enzymes critical for ECM degradation [69]. KLF10 has been shown to modulate TGF-β signaling and influence fibroblast function and extracellular matrix production, suggesting involvement in wound healing [70]. Notably, KLF10 and KLF6 act as negative regulators of scar formation by inhibiting fibroblast proliferation and collagen synthesis [28]. Restoring the activity of these factors could help reduce excessive scar formation

## 4. KLFs in Gastrointestinal Cancer

KLFs are frequently dysregulated in human GI cancers and can function either as tumor suppressors or oncogenes depending on cellular context, tissue type, and genetic background. They also engage in functional crosstalk with pivotal oncogenes and tumor suppressors such as p53, KRAS, APC, and PTEN. Identified functions of the KLFs in GI cancers are detailed in Table 2 and will be outlined by tissue type below.

## 5. KLFs in Esophagus

### 5.1. Squamous Epithelium

The stratified squamous epithelium of the esophagus relies on a finely tuned balance between proliferation and differentiation, with proliferating cells in the basal layer that differentiate as they migrate through the suprabasal and superficial layers [15,71,72]. A long non-coding RNA targeting KLF3 inhibits esophageal squamous cell cancer (ESCC) migration and invasion [73]. KLF4 and KLF5 have distinct and often opposing roles in regulating proliferation and migration in normal esophagus. KLF4 is typically expressed in suprabasal epithelial cells, where it promotes terminal differentiation and helps maintain epithelial barrier function, while KLF5 is predominantly enriched in basal cells [74]. In mice, esophageal deletion of *Klf4* delays epithelial differentiation and leads to squamous cell dysplasia [17]; KLF4 appears to promote differentiation and stratification in the esophagus by activating *Wnt5A* [75]. Interestingly, overexpression of *Klf4* in the esophageal epithelium also disrupts homeostasis but in this case by activating pro-inflammatory pathways [54], and KLF4 activates NFκB signaling via RHOF [76]. KLF4 also exhibits a stage-specific role in ESCC; it is frequently downregulated in high-grade dysplasia and early-stage disease, suggesting a tumor-suppressive role during early carcinogenesis, but its expression increases in advanced stages of ESCC, where it appears to contribute to tumor progression [77]. KLF5 promotes proliferation and migration of esophageal squamous epithelial cells via MERK/ERK and the integrin-linked kinase, respectively, while long-term loss of KLF5 function in squamous epithelial cells induces epithelial-to-mesenchymal transition (EMT), characterized by reduced proliferation but increased cell migration and invasive capabilities; in the context of mutant p53, *KLF5* loss leads to the development of ESCC [74,78,79,80]. KLF5 is upregulated in inflammatory esophagitis and responds to pro-inflammatory cytokines such as interleukin-1β (IL-1β) and tumor necrosis factor-α (TNF-α) [81]. Interestingly, in the presence of mutant p53, KLF5 may exert anti-tumorigenic effects by upregulating the cyclin-dependent kinase inhibitor p21, highlighting its dual role depending on the cellular context [82]. In addition, forced re-expression of KLF5 in ESCC cells triggers apoptosis via BAX and JNK activation, yet KLF5 suppresses ferroptosis, and genetic loss or degradation by NEDD4L or promotes ferroptotic cell death, sensitizing tumors to radiotherapy [83,84]. Thus, the role of KLF5 in ESCC is complex. In contrast, KLF6 more consistently demonstrates tumor-suppressive activity in ESCC. For instance, miR-498 activates the FOXO1/KLF6 transcriptional axis, thereby alleviating tumor progression [85], and KLF6 also directly binds miR-4262 to inhibit ESCC development [86].

Other KLFs play important roles in the esophagus and particularly in ESCC. KLF9 is markedly downregulated in ESCC, inversely associated with aggressive clinical features, and inhibits tumor growth, migration, and metastasis by binding TCF4 and repressing β-catenin/TCF signaling [87], and hypoxic cells release exosomes rich in miR-340-5p, which targets KLF10 in normoxic cells to promote radioresistance [88]. In ESCC, KLF12 downregulation drives cisplatin resistance and metastasis by derepressing L1CAM, a process modulated by TRIM27-mediated ubiquitination [89]. KLF13 promotes ESCC cell proliferation, migration, EMT, and tumor growth, both driving tumor progression and modulating lipid metabolism by positively regulating GPIHBP1 [90].

### 5.2. Barrett’s Esophagus

Barrett’s esophagus (BE) is a pre-neoplastic condition of the lower esophagus associated with chronic exposure to refluxate [91]. In this condition, the normal stratified squamous epithelium is replaced by metaplastic columnar epithelium, and BE can progress to esophageal adenocarcinoma (EAC). While its overall expression remains relatively unchanged, KLF5 undergoes chromatin redistribution to activate an EAC-associated cell cycle gene programs [92]. Similarly, KLF4, is upregulated in response to acid reflux via NF-κB signaling, and KLF4 and CDX2 mutually regulate each other at the transcriptional level, promoting mucin (MUC2) production-a hallmark of intestinal metaplasia. Furthermore, KLF4 overexpression can amplify NF-κB signaling through RhoF, establishing a pro-inflammatory feedback loop that facilitates the development of BE [93]. In addition, reflux-induced prostaglandin E2 (PGE2) synthesis, driven by cytosolic phospholipase A2 (cPLA2), is transcriptionally regulated by SP/KLF family complexes. Among these, KLF11 acts as a negative regulator by repressing cPLA2 expression, thereby modulating the rate-limiting step in PGE2 biosynthesis and potentially limiting chronic inflammation [94]. Meanwhile, KLF6 is downregulated in BE, although its precise role in metaplastic transformation and progression to EAC remains largely unexplored [95].

## 6. KLFs in Stomach

The gastric epithelium is a continuously self-renewing tissue sustained by stem cells located within the gastric glands, with daughter cells migrating bidirectionally as they differentiate [96]. Expression of the KLFs in the stomach is spatially regulated, reflecting their region-specific functions in epithelial maintenance and transformation [10]. Both KLF4 and KLF5 have been extensively studied in the context of gastric epithelial biology and cancer. KLF4 is predominantly expressed in the mid to upper regions of the gastric epithelium, particularly within the differentiated surface epithelial cells of the fundus and body, and loss of *Klf4* mice results in a type of precancerous gastric metaplasia known as spasmolytic polypeptide-expressing metaplasia (SPEM) [20,97]. In gastric cancer, KLF4 expression is progressively reduced during gastric cancer development and progression, and KLF4 loss is associated with increased epithelial proliferation and a worse prognosis [98,99], with higher cytoplasmic KLF4 expression linked to improved overall survival in gastric patients [100]. KLF4 overexpression in gastric cancer cells reduces colony formation, migration, invasion, and tumor growth, while also promoting apoptosis and cell cycle arrest [101]. In contrast, KLF5 is predominantly expressed within the isthmus and neck regions of the gastric glands, which are rich in proliferating progenitor cells [102]. This localization aligns with its putative oncogenic function, as KLF5 promotes gastric epithelial proliferation and is frequently elevated in poorly differentiated gastric tumors and advanced TNM stage [103,104]. KLF5 loss disrupts cell cycle progression by upregulating p21 and CDK4 and inducing G0/G1 arrest [105], and KLF5 also regulates cancer-associated fibroblasts to promote tumor cell migration through CCL5–CCR5 signaling, underscoring a role of KLF5 in tumor–stroma crosstalk [106].

Several other KLF members, including KLF1, KLF2, KLF3, KLF6, KLF16, and KLF17, are broadly expressed in the stomach although detailed region-specific expression data for these factors remain limited. KLF2, KLF6 and KL17 are generally present in normal gastric tissues and downregulated in gastric cancer, suggesting potential tumor-suppressive roles [107,108,109,110,111], whereas KLF1, KLF3, and KLF16 are elevated in gastric cancer; KLF1 and KLF3, in particular, activate the WNT/β-catenin pathway suggesting an oncogenic function [112,113,114]. Surprisingly, diffuse-type gastric cancers with high KLF2 expression are associated with the presence of plasma cells and poor prognosis, highlighting the need to fully understand KLF functions in different cancer types, even within the same tissues, and within the tumor microenvironment [115]. In gastric cancer, KLF6 exerts a tumor-suppressive role by inhibiting proliferation through transcriptional upregulation of p21 and repression of the oncogene c-MYC [107], while KLF7 is markedly overexpressed, promotes tumor progression by enhancing gastric cancer cell migration, and is associated with advanced TNM stage, lymphovascular invasion, and reduced survival [116]. KLF8 is induced in gastric cancer cells by TGF-β1 and hypoxia and promotes EMT, migration, invasion, glycolysis via GLUT4, and multidrug resistance through MDR1, Bcl-2, and P-gp [117,118,119,120]. KLF9 suppresses gastric cancer metastasis by repressing MMP28 transcription, but KLF9 also mitigates platinum resistance in gastric cancer, highlighting its complex roles in gastric cancer and importance of context [121,122]. While KLF10 is downregulated in gastric cancer, with its loss correlating with advanced stage and poor prognosis, [123], KLF11 is upregulated in gastric cancer tissues and cell lines, promoting cell invasion and migration primarily by increasing Twist1 expression [124].

In poorly differentiated gastric cancer, KLF12 acts as a potential oncogene, where knockdown induces growth arrest and alters proliferation-related genes and overexpression enhances invasiveness [125]. KLF13 lacks a clearly defined spatial expression pattern in the normal stomach but is upregulated in gastric tumors [126]. High KLF13 expression is associated with poor clinical outcomes, knockdown of KLF13 in gastric cancer cells significantly impairs migration and invasion, and KLF13 promotes tumor progression by activating NF-κB signaling to enhance migration, invasion, inflammation, and metastasis, and enhances gastric cancer cell by activating the NF-κB pathway [126]. In contrast, KLF15 appears to function as a tumor suppressor [127]. KLF15 expression is downregulated in gastric cancer and inversely correlates with clinical stage, lymphatic invasion, and distant metastasis. Mechanistically, KLF15 suppresses cancer cell proliferation by upregulating CDKN1A/p21 and CDKN1C/p57, two key inhibitors of the cell cycle, and suppresses migration by upregulating the lncRNA TFAP2A-AS1 [128,129]. KLF17 expression is diminished in both gastric cancer tissues and cell lines, correlating with larger tumor size, invasion, lymph node metastasis, and advanced TNM stage, and restoring KLF17 in these cells enhances the sensitivity to 5-fluorouracil by suppressing key mediators of chemoresistance, including P-glycoprotein, MRP1, and BCL-2 [130].

## 7. KLFs in Intestine

The intestinal epithelium is rapidly renewing and maintains homeostasis through a tightly regulated balance between stem cell proliferation in crypts and terminal differentiation along the villus axis in the small intestine and the upper crypt in the colon [131]. This renewal process is governed by precise transcriptional programs that preserve epithelial integrity, facilitate nutrient absorption, and sustain barrier function [132,133]. Within the intestine, the KLFs orchestrate epithelial dynamics, stress responses, and tumorigenesis, as well as metabolism and particularly bile acid homeostasis. Notably, KLF4, KLF5, KLF9, and KLF15 have emerged as central modulators of intestinal physiology [10]. KLF4 and KLF5 exhibit reciprocal expression patterns that define distinct epithelial compartments. KLF5 is enriched in proliferative crypt cells, where it promotes stem cell renewal via WNT/β-catenin signaling and repression of Sox9 [134,135], while KLF4 is predominantly expressed in villus and goblet cells, driving terminal differentiation, barrier formation, and mucin production by modulating BMI1^+^ intestinal stem cell fate [22]. Loss of *Klf5* in mice disrupts crypt architecture and compromises barrier integrity, partly through downregulation of desmoglein-2 [136]. Conversely, *Klf4* deficiency impairs goblet cell differentiation, localization of Paneth cells via Ephrin-B1 suppression, and weakens barrier defense [23,137].

KLFs also integrate stress and injury signals into intestinal epithelial repair programs, play essential roles in intestinal absorption, and are important mediators of intestinal cancers. KLF4 is a key mediator of the DNA damage response, induced in a p53-dependent manner to enforce G_1_/S cell cycle arrest via p21 (CDKN1A) upregulation [138]. Depending on context, KLF4 may also suppress apoptosis and inflammation in intestinal ischemia–reperfusion injury via GPR30 [139]. In injury models, KLF4 supports reserve stem cell function, limits NF-κB–driven inflammation and promotes regeneration [22,55]. Mechanistically, KLF4 antagonizes WNT signaling, regulates goblet cell markers (MUC2, CA1), and interacts with Notch and YAP/TAZ pathways to influence lineage fate [23,140]. KLF4 directly regulates the expression of nutrient transporters involved in zinc and biotin uptake, with loss also leading to downregulation solute carrier (SLC) family transporters, highlighting its role in maintaining epithelial health and absorptive capacity [141,142,143]. In *Apc*^Min^ intestinal tumorigenesis models, mice with *Klf4* haploinsufficiency develop ~50% more adenomas than mice with *Apc*^Min^ alone, with *Klf4* mRNA levels inversely correlating with adenoma size [144]. Overexpression of KLF4 also suppresses CRC cell proliferation, migration, and invasion, while reduced KLF4 expression is associated with ulcerative colitis and sporadic colorectal adenomas [145,146,147]. In addition, exosomal miR-25-3p targets KLF4, as well as KLF2, to promote vascular permeability and angiogenesis and enhances CRC metastasis [148]. In contrast, KLF5 promotes proliferative repair responses by activating Lgr5, Ascl2, and IL-22/JAK2/STAT3 signaling pathways [149,150]. Conditional deletion of KLF5 in intestinal epithelial cells demonstrates its role in proliferation, differentiation, and spatial organization along the crypt radial axis [134]. Intestinal deletion of *Klf5* is perinatally lethal, and conditional knockout in adults impairs regenerative capacity despite partial compensation by Sox9 and Reg family genes [135]. KLF5 is also a putative oncogene in CRC as upregulation promotes tumorigenesis through oncogenic signaling [151,152,153]. Interestingly, metabolic modulation reveals an Achilles’ heel for KLF5-driven tumors; ketogenic interventions, either with β-hydroxybutyrate supplementation or overexpression of the ketogenic enzyme HMGCS2, markedly downregulate KLF5, thereby suppressing CXCL2 expression in cancer-associated fibroblasts (CAFs). This shift reprograms the tumor immune microenvironment, reducing immunosuppressive cell infiltration and enhancing cytotoxic NK and T cell recruitment in CRC models. Furthermore, pharmacologic inhibition of KLF5 or dietary ketogenic regimens restore oxaliplatin sensitivity in CRC patient-derived organoids by simultaneously inhibiting the BCL2/caspase-3 survival pathway and triggering ferroptosis via the LIF/MTF1/FPN1 axis [154,155,156].

Among the other KLFs, KLF1 expression is elevated in colorectal cancers [112], while KLF3 is targeted by miR-425 to promote CRC cell proliferation and metastasis [157]. In the intestinal epithelium, a specialized subset of T cells derived from TH17 cells can initiate spontaneous epithelial transformation through a KLF6 and T-BET dependent interferon-γ program that operates independently of IL-17; this tumorigenic cascade is normally kept in check by epithelial-derived TGFβ1 signaling, which inhibits KLF6-driven T-BET expression [158]. KLF7 is overexpressed and facilitates cell invasion, migration, and tumor growth by directly binding to the miR-139-5p promoter, and silencing KLF7 restores miR-139-5p and suppresses CRC progression [159]. KLF7 binds to the PDGFB promoter, enhancing its transcription and secretion, and secreted PDGFB activates downstream MAPK/ERK, PI3K/AKT, and JAK/STAT3 [160]. In colorectal cancer, KLF8 enhances EMT and metastasis by activating FHL2 [161]. Jejunum-specific deletion of KLF9 results in shorter villi, reduced crypt proliferation, decreased goblet cells, and increased Paneth cell numbers [37]. Loss of KLF9 in *Apc*^Min^ mice increases colon adenomas via upregulation of interferon-stimulated genes such as ISG15, which inhibit apoptosis and reduce 5-fluorouracil sensitivity [162,163]. KLF9 directly represses ISG15 to limit tumorigenesis, and human CRC has significantly lower KLF9 mRNA and protein levels compared with normal mucosa, underscoring its tumor-suppressive role. Nutrient entry into the duodenum triggers cholecystokinin (CCK)-mediated bile acid release from the gallbladder, and bile acids then establish a negative feedback loop involving the nuclear receptor FXR, FGF15, and hepatic cholesterol 7α-hydroxylase (Cyp7a1) [164]. KLF9 contributes to bile acid reabsorption by regulating apical sodium-dependent bile acid transporter (Asbt) expression in the ileum [165]. Reabsorbed bile acids activate FXR/Fgf15 signaling, and KLF9-deficient mice exhibit elevated bile acid levels in the gallbladder and feces, with reduced serum bile acid concentrations.

In rectal cancer, KLF12 is associated with disease-free survival, immune infiltration, and immune checkpoint expression, and KLF12 overexpression inhibits proliferation, migration, and invasion [166]. KLF14 downregulation in CRC correlates with advanced tumor stage, larger tumor size, and worse overall disease-free survival [167,168]. Loss of KLF14 induces centrosome overduplication via Plk4, leading to spontaneous tumorigenesis and colon cancer progression, while KLF14 overexpression induces mitotic catastrophe and reduces glycolysis by downregulating the glycolytic enzyme LDHB. Like KLF9, KLF15 is also a key regulator of bile acid synthesis. KLF15 represses intestinal Fgf15 and sustains hepatic Cyp7a1, the rate-limiting enzyme for bile acid synthesis, to maintain bile acid pools, and *Klf15* loss reduces bile acid levels and impairs lipid absorption, effects that are reversible by ileectomy [38,169]. Importantly, KLF15 expression is under circadian control, linking transcriptional regulation to diurnal bile acid rhythms [170]. In CRC, KLF15 appear to be tumor suppressive and activates the lncRNA LINC00689, which recruits PTBP1 to stabilize LATS2 mRNA, thereby inhibiting the YAP1/β-catenin pathway and suppressing proliferation and metastasis [171]. Conversely, KLF16 is elevated in CRC and associated with poor prognosis; under endoplasmic reticulum (ER) stress, KLF16 translocated to the nucleolus and interact with NPM1 and FBL, two essential proteins for nucleolar homeostasis, to promote translational reprogramming and enhance the stress tolerance of CRC cells [172]. KLF17 expression is reduced in CRC, and downregulation, driven largely by promoter hypermethylation, is associated with lymph node metastasis and poor overall survival, while positive expression in stage III CRC patients correlates with significantly longer disease-free survival [173,174]. Functionally, KLF17 transcriptionally activates FHL1, leading to increased E-cadherin and decreased N-cadherin and Vimentin expression and reduced tumor growth [175] and mediates the antitumor effects of PX-12, a thioredoxin-1 (Trx-1) inhibitor, in CRC [176].

## 8. KLFs in Pancreas and Liver

**Figure 3:**
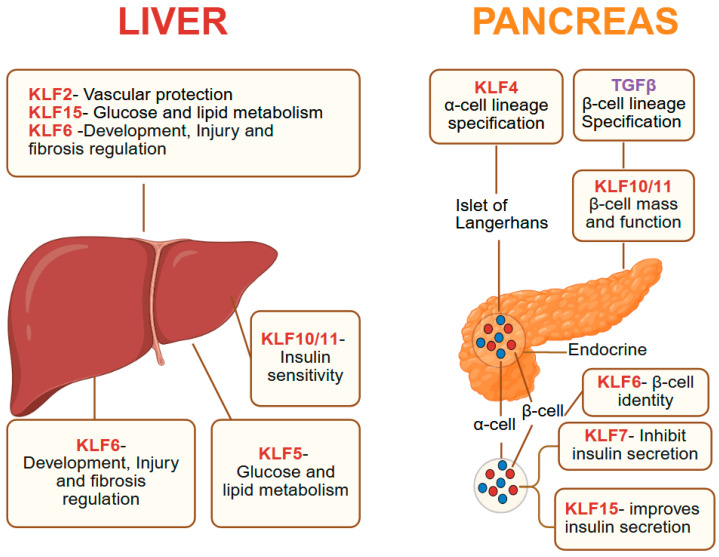
KLFs in hepatic and pancreatic physiology. In the liver, KLFs modulate metabolic homeostasis by regulating genes involved in carbohydrate, lipid, and protein metabolism. KLF15 participates in gluconeogenesis, lipid oxidation, and amino acid metabolism; KLF10 and KLF11 influence hepatic insulin sensitivity and TGFβ-mediated pathways; KLF6 is involved in liver development, injury response, and fibrosis regulation. In the pancreas, KLF10 and KLF11—downstream targets of TGFβ—are enriched in islets of Langerhans and regulate β-cell mass, insulin gene expression, and β-cell function via SERTA Domain-Containing Protein 1 (SEI1) and pancreatic-duodenal homeobox-1 (Pdx1). KLF6 maintains β-cell identity by upregulating β-cell–specific genes (Ins1, Ins2, Pdx1, Mafa) and preventing β-to-α transdifferentiation through meteorin-like (METRNL), whereas KLF7 impairs insulin secretion and β-cell gene expression while promoting inflammation (IL-6). KLF4 reprograms acinar cells toward ductal cell lineages and, in cooperation with NKX2.2, regulates α-cell–specific gene signatures. KLF15 improves insulin secretion and confers resistance to diet-induced obesity by suppressing stearoyl-CoA desaturase 1 (SCD1). Created with BioRender.com.

Across the liver and pancreas, KLF family members coordinate key aspects, including metabolism, cell identity, regeneration, and injury response (Figure 3). Broadly, KLF1 is central to fetal liver erythropoiesis, KLF2 and KLF6 modulate vascular and inflammatory responses, KLF4, KLF5, and KLF6 regulate regeneration and cell fate decisions, and KLF10, KLF11, and KLF15 govern metabolic pathways. Dysregulation of these factors contributes to metabolic disorders, organ injury, and altered cellular plasticity, making them promising targets for therapeutic intervention in the liver and pancreas.

### 8.1. Pancreas

The pancreatic parenchyma contains both exocrine and endocrine components; the exocrine pancreas comprises acinar and ductal cells, whereas the endocrine compartment consists of the islets of Langerhans [33]. *Klf4* overexpression in pancreatic acinar cells represses acinar identity and promotes ductal lineages, while pancreas-specific *Klf4* ablation inhibits the formation of pancreatic intraepithelial neoplasia (PanIN), a precursor of pancreatic ductal adenocarcinoma (PDAC), induced by mutant *Kras*^G12D^ [14]. KLF5 expression is high in the embryonic pancreas, decreases postnatally, and is elevated in pancreatic cancers [177], and deletion of *Klf5* in acinar cells in mice increases expression of the tumor suppressor NDRG2 and reduces activation of STAT3, leading to a reduction in acinar-to-ductal metaplasia and PanINs [13]. KLF6 supports β-cell identity by upregulating INS1, INS2, PDX1, and MAFA, and preventing β-to-α transdifferentiation through METRNL [178,179]. Physiological stimuli such as hyperglycemia, obesity, and pregnancy promote β-cell proliferation, while pathological conditions like type II diabetes trigger transdifferentiation, which KLF6 counteracts. In PDAC, KLF8+/vimentin+ circulating tumor cells predict higher relapses and poor prognosis [180]. KLF10 maintains pancreatic function via SERTA domain-containing protein 1 (SEI1) and regulates β-cell mass [181], and KLF11 is essential for pancreatic organogenesis and adult β-cell maintenance, partly via Pdx1 activation [182]. In PDAC cells, KLF10 suppresses the pro-metastatic effects of TGFβ [183]. These studies highlight important functions for KLFs in pancreatic development, cell identity, and neoplastic progression.

### 8.2. Liver

The liver plays a central role in metabolic homeostasis and is essential for detoxification, the synthesis and degradation of carbohydrates, fats, and proteins, maintaining blood glucose balance, and immune responses [184]. Several members of the KLF family are critical regulators of these processes. KLF1 has a tightly regulated spatiotemporal pattern during erythroid development, appearing first in primitive erythroid cells (E7.5) and later in hepatic primordia (E9), with sustained high expression in fetal liver until at least E14.5 [185]; while its essential role in fetal liver erythropoiesis is established, broader hepatic functions remain unclear. KLF2 is vasoprotective and is activated by simvastatin and resveratrol during liver reperfusion, enhancing autophagy and reducing inflammatory cytokines such as IL-1, IL-6, and TNFα [186,187]. However, endothelial *Klf2* deletion reduces injury and promotes proliferation in chronic CCl_4_-induced damage by inducing activin A expression [188], and KLF2 overexpression promotes steatosis via SCAP/SREBP1-mediated lipogenesis [189]. Mice lacking KLF3 have smaller adipocytes, reduced white adipose tissue, and resistance to obesity and glucose intolerance, suggesting a protective role against hepatic steatosis [190,191]. The liver regenerates efficiently after injury or partial resection, and hepatocyte-specific expression of KLF4, along with SOX2, OCT4, and C-Myc (4F) reprograms adult hepatocytes, triggering dedifferentiation followed by proliferation [192]. KLF5 is enriched in and regulates ductular reaction with dynamic tissue expansion and remodeling critical for regeneration; while deletion *Klf5* in all liver cells produces no phenotype under normal conditions, *Klf5* loss specifically in biliary epithelial cells impairs biliary remodeling with cholestatic liver injury in mice [193]. KLF6 is essential for liver development as *Klf6* null mice die by embryonic day 12.5 due to failed liver formation and loss of endoderm markers [194,195]. In adult hepatic ischemia–reperfusion injury, KLF6 is upregulated, and its loss exacerbates injury, apoptosis, and inflammation, while KLF6 overexpression is protective [196]. KLF6 is generally tumor-suppressive in hepatocellular carcinoma (HCC), but the tumor microenvironment can subvert these tumor-suppressive effects by creating a self-perpetuating oncogenic feedback loop in which tumor-associated macrophages and the oncogenic epigenetic regulator UHRF1 cooperate to suppress KLF6, sustain immune evasion, and drive HCC progression [197].

KLF7 is broadly oncogenic, and in HCC, KLF7 overexpression promotes inflammation-driven metastasis by transactivating TLR4 and PTK2, forming a positive feedback loop with HMGB1; KLF7 depletion significantly impedes HCC progression [198]. KLF10 and KLF11 are both downstream targets of TGFβ and act as negative regulators of gluconeogenesis. KLF10 is induced by carbohydrate stimulation and suppresses carbohydrate response element binding protein (ChREBP) target genes, forming a feedback inhibition loop; its deletion downregulates glycolytic genes and upregulates gluconeogenic and lipogenic programs, while KLF11 represses Pck1 (PEPCK), a key gluconeogenic enzyme [199,200,201]. KLF15 links glucose and lipid metabolism, as fasting induces KLF15, which promotes gluconeogenesis and suppresses lipogenesis through recruitment of the corepressor RIP140 to the Srebf1 promoter in complex with LXR/RXR [202]. Liver-specific *Klf15* ablation in diabetic mice downregulates gluconeogenesis and amino acid catabolism genes, improving hyperglycemia [203]. Metformin lowers blood sugar partly via KLF15 downregulation, and in diabetes, reduced degradation by the E3 ligase WWP1 elevates KLF15, driving muscle atrophy via atrophy-related gene induction [204]. In adipose tissue, KLF15 overexpression suppresses lipogenesis through PPARγ activation, whereas its loss promotes fatty liver development [205].

## 9. KLFs in Signaling Pathways of the Gastrointestinal Tract

The expression of KLFs in the gastrointestinal tract is highly dynamic and responsive to diverse physiological and pathological stimuli [1,2], and the KLFs play multifaceted regulatory roles in diverse signaling cascades, including PI3K/AKT, NF-κB, Wnt/β-catenin, Notch, TGF-β, and MAPK. These pathways are highly interconnected and can activate or suppress one another. Through modulation of these signaling networks, KLFs influence a broad spectrum of biological processes. Notably, KLFs do not merely act as downstream targets of these pathways but also actively modulate their activity in a context- and region-specific manner [3]. This section of the review focuses on the intricate interplay between KLFs and these major signaling cascades, with particular attention to the bidirectional interactions in which KLFs and signaling pathways mutually regulate each other. Special emphasis is placed on pathways frequently dysregulated in GI disorders including cancer, highlighting the pivotal role of KLFs in their regulation.

### 9.1. PI3K/AKT Signaling

**Figure 4:**
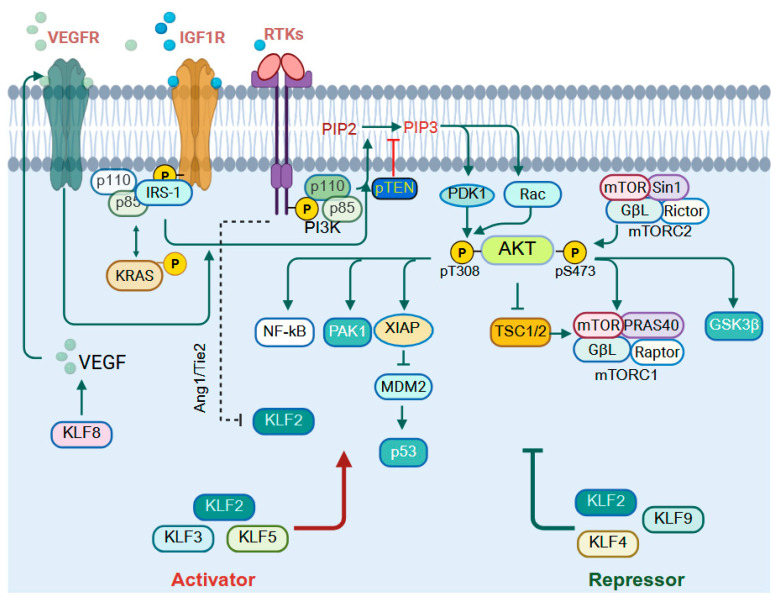
KLFs in PI3K/AKT signaling. PI3Ks are classified into three types: class I, II, and III, based on the triggering stimulus. The pathway initiates with the phosphorylation of PIP2 to PIP3, a second messenger that activates various substrates. KLF2 exhibits context-dependent regulation of PI3K/AKT signaling. it suppresses AKT activity in colorectal cancer by downregulating GPX4 to induce ferroptosis, yet in pancreatic β-cell injury, resveratrol-induced KLF2 activates PI3K/AKT via miR-126. KLF3 enhances AKT signaling in hepatocytes through miR-21-5p–mediated control of lipogenesis, whereas KLF4 acts oppositely, repressing AKT2 via miR-206 in gastric cancer and reducing AKT activity under MoS_2_-induced stress, thereby impairing intestinal morphology. KLF5 demonstrates strong reciprocal crosstalk with AKT: it promotes hepatocellular carcinoma progression via the PI3K/AKT/Snail axis. In turn, AKT stabilizes KLF5 by blocking GSK3β-mediated degradation, while a P301S mutation in colon cancer further prevents degradation, sustaining oncogenic KLF5 and reinforcing a feed-forward loop with AKT. KLF9 suppresses PI3K/AKT signaling. Several KLFs, indicated at the bottom of the figure, can either activate or repress the pathway, but the mechanisms of this regulation are not clear. (Activation is represented by a pointed arrow and repression by a flathead arrow). Created with BioRender.com.

The PI3K/AKT pathway controls many aspects of cell function, such as growth, survival, and metabolism, responding to signals like growth factors, hormones, and nutrients [206]. Broadly, KLFs can function as either activators (KLF1, KLF5, KLF6, KLF8, KLF9, KLF14, and KLF15) or repressors (KLF2, KLF3, KLF4, KLF10, and KLF11) of PI3K/AKT signaling, though some exhibit context-dependent roles (Figure 4). KLF2 demonstrates a dual regulatory role in PI3K/AKT signaling whereby in colorectal cancer, ectopic overexpression of KLF2 reduces tumorigenicity by inducing ferroptosis through downregulation of GPX4, thereby suppressing PI3K/AKT activity [207], while in pancreatic β-cell injury caused by uric acid, resveratrol upregulates KLF2, which in turn modulates miR-126 to activate PI3K/AKT signaling [208]. KLF3 enhances PI3K/AKT signaling; in hepatocytes, miR-21-5p regulates lipogenesis and oxidative stress by targeting KLF3, which in turn activates the PI3K/AKT pathway [209]. In contrast, KLF4 inhibits this pathway by upregulating miR-206, which directly targets AKT2 in gastric cancer [210]. KLF5 and AKT exhibit reciprocal crosstalk that profoundly influences GI tumorigenesis. In hepatocellular carcinoma, KLF5 promotes tumor growth and metastasis through activation of the PI3K/AKT/Snail axis [211], and in colorectal cancer, KLF5 activates PI3K/AKT signaling to reprogram fatty acid metabolism and reduces immune cell infiltration, ultimately fostering therapy resistance [212].

In normal keratinocytes under stress, KLF5 and p53 together transactivate AKT1 and AKT3, diverting cells toward survival pathways [8]. The interaction between KLF5 and AKT is not unidirectional, as AKT can stabilize KLF5 through indirect inhibition of GSK3β, which normally phosphorylates KLF5 at serine 303 (S303) to promote its degradation [152]. Importantly, in colon cancer, a somatic P301S mutation prevents GSK3β-mediated phosphorylation at S303, resulting in constitutive KLF5 stabilization, extended half-life, and increased transcriptional activity. This suggests a feed-forward regulatory loop, wherein KLF5 activation of PI3K/AKT signaling enhances AKT activity, which in turn sustains and potentiates KLF5, amplifying its oncogenic influence. Fatty acid binding protein 5 (FABP5) enhances CREB phosphorylation, which transcriptionally induces miR-889-5p expression to suppress KLF9, thereby activating PI3K/AKT signaling and driving HCC progression [213]. Future research on this KLF/PI3K/AKT axis could pave the way for novel therapeutic strategies targeting this pathway.

### 9.2. NF-kB Signaling

**Figure 5:**
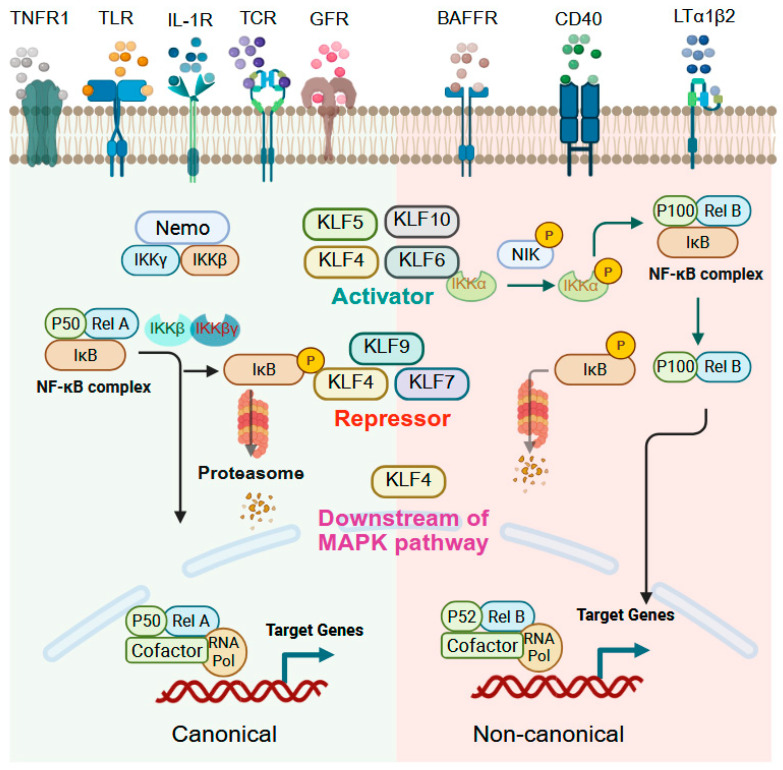
KLFs in NFkB signaling. NF-κB signaling is modulated by diverse stimuli including cytokines, pathogens, and stress signals. In its inactive state, NF-κB dimers are sequestered in the cytoplasm by IκB proteins; upon activation, IκBs are phosphorylated and degraded, allowing NF-κB nuclear translocation and target gene transcription. KLF4 can both activate and repress NF-κB: in ESCC and eosinophilic esophagitis, KLF4 promotes NF-κB via RhoF upregulation, whereas steatotic hepatocyte-derived extracellular vesicles suppress endothelial KLF4, enhancing NF-κB-mediated vascular inflammation. In hepatocytes, Kallistatin-induced KLF4 activates NF-κB by preventing p65 sequestration, while DSS-induced colitis is reduced in KLF4-deficient mice, highlighting KLF4′s essential role in intestinal inflammation. KLF5 and KLF6 act as NF-κB activators, promoting inflammation in epithelial and myeloid cells, whereas KLF9 and KLF10 function as suppressors. Created with BioRender.com.

Nuclear factor kappa-B (NF-κB) comprises a family of transcription factors, including includes p50/p105/NF-κB1, p52/p100/NF-κB2, Rel/c-Rel, p65/RelA, and RelB, that orchestrate inflammation, immunity, cell survival, and apoptosis by responding to a wide range of stimuli such as pro-inflammatory cytokines, pathogens, oxidative stress, and environmental stressors [214,215,216]. The role of KLFs in NF-κB signaling is highly context-dependent, functioning either as activators or repressors depending on cell type, stimulus, and disease state (Figure 5). KLF4, for example, shows bidirectional regulation of NF-κB. In ESCC and eosinophilic esophagitis (EoE), KLF4 promotes NF-κB activation through upregulation of RhoF, a GTP-binding protein that stimulates NF-κB signaling [76]. Conversely, in non-alcoholic fatty liver disease, extracellular vesicles enriched in microRNA-1 suppress KLF4 in endothelial cells, thereby enhancing NF-κB activation and vascular inflammation [217]. In hepatocytes, Kallistatin induces KLF4, which in turn activates NF-κB by downregulating CGI-58, preventing sequestration of the p65 subunit [218]. Similarly, DSS-induced colitis is attenuated in *Klf4*-deficient mice, demonstrating the necessity of KLF4 for NF-κB pathway activation in intestinal inflammation [55]. Interestingly, KLF4 itself can also be downstream of NF-κB; in gastric intestinal metaplasia, KLF4 is induced via NF-κB signaling activated by hTERT [219]. However, in hepatocellular carcinoma (HCC), KLF4 is ubiquitinated and degraded by TRAF7, a TNFα/NF-κB pathway E3 ubiquitin ligase, leading to tumor-promoting effects [220]. Both KLF5 and KLF6 can activate NF-kB signaling in the intestine. LPS exposure to intestinal epithelial cells induces inflammation through KLF5-mediated transcriptional upregulation of p50 and p65 [221]. KLF6, which is highly expressed in myeloid cells and inflamed intestinal tissue from IBD patients, regulates macrophage responses by promoting NF-κB activation while suppressing STAT3 signaling. Loss of KLF6 in mouse intestine confers resistance to colitis, underscoring its role in intestinal inflammation [31]. In contrast, KLF9 and KLF10 function as suppressors of NF-κB. Arsenic-induced hepatic toxicity downregulates KLF9, which normally serves as a positive regulator of PPARγ, a transcription factor that inhibits NF-κB directly or indirectly by stabilizing the NF-κB inhibitor IκB [222]. KLF10; meanwhile, restrains NF-κB activity in pancreatic cancer by maintaining SIRT6 expression, which suppresses p65 phosphorylation [223]. In summary, NF-κB and KLFs represent critical, interconnected transcriptional regulators of inflammation and disease progression.

### 9.3. Wnt Signaling

**Figure 6:**
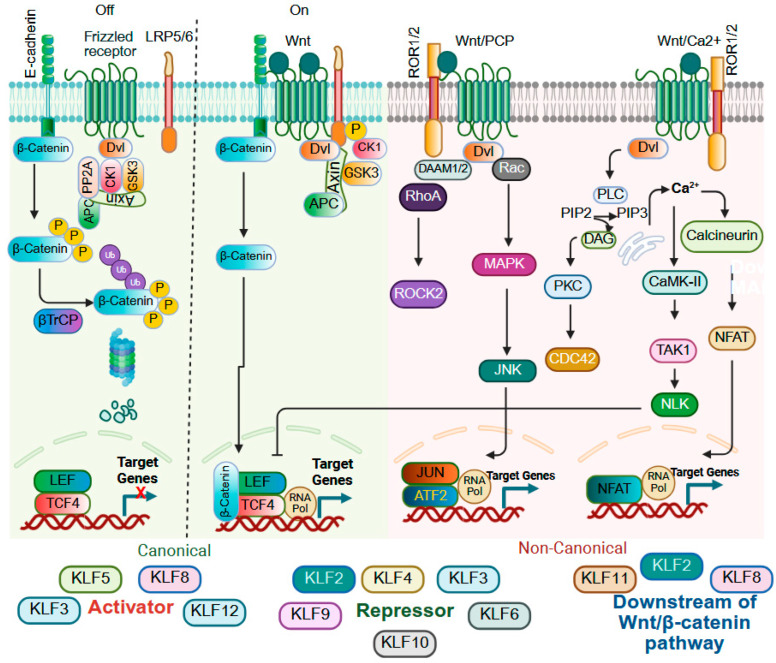
KLFs in WNT signaling. WNT signaling is classified into canonical and non-canonical pathways. The canonical pathway operates through the stabilization and nuclear translocation of β-catenin whereas non-canonical Wnt signaling executes its action independently of β-catenin and relies on calcium signaling as a central mediator. The roles of KLFs in modulating Wnt/β-catenin signaling is complex and context dependent. KLF1 and KLF3 act upstream of Wnt/β-catenin to promote EMT in gastric cancer, while loss of KLF3 enhances Wnt1 and β-catenin activity. KLF2 functions as a downstream effector of Wnt signaling in pluripotent stem cells but inhibits Wnt activity in a mutant p53–dependent manner. KLF4 suppresses Wnt signaling by preventing β-catenin acetylation and nuclear translocation, whereas KLF5 enhances β-catenin/TCF4 transcriptional activity and promotes proliferation. KLF6 and KLF10 generally repress Wnt signaling and stemness, although under chemotherapy resistance conditions KLF6 can cooperate with TAF6L to enhance nuclear β-catenin. KLF8 acts both downstream and upstream of Wnt/β-catenin. KLF9 inhibits Wnt signaling via downregulation of Frizzled-5, KLF11 cooperates with β-catenin to promote stemness genes. KLF12 activates Wnt signaling through DVL2 induction. KLFs exert oncogenic or tumor-suppressive effects on Wnt signaling in a highly context-dependent manner. Created with BioRender.com.

Wingless/integrated protein (Wnt) signaling is essential for embryonic development, including body axis formation, and adult tissue homeostasis and regeneration, including cell fate determination, cell migration and polarity, and stem cell maintenance, and dysregulation of Wnt signaling is strongly associated with human diseases, particularly cancer [224,225]. The Wnt family comprises secreted glycoproteins that bind to cell surface receptors and activate downstream signaling through two major pathways: canonical Wnt/β-catenin signaling and non-canonical pathways, the latter of which operate independently of β-catenin and frequently rely on calcium signaling or cytoskeletal regulation [226,227,228]. KLFs are pivotal regulators of Wnt signaling, acting as either activators or repressors depending on tissue or cellular context, and their influence extends beyond direct modulation of canonical or non-canonical cascades to also regulate expression of Wnt ligands, receptors, and antagonists (Figure 6). In gastric cancer KLF1 and KLF3 activate canonical Wnt/β-catenin signaling to promote EMT, but in colorectal cancer, reduced KLF3 expression promotes Wnt1 accumulation and activation of β-catenin signaling [113,114,229]. KLF2 also demonstrates dual functionality. In pluripotent stem cells, KLF2 functions downstream of Wnt/β-catenin signaling and cooperates with Tfcp2l1 to establish and maintain naïve pluripotency [230], while in colon cancer, simvastatin induces KLF2 expression in a mutant p53-dependent manner, leading to upregulation of p21^WAF1/CIP1^ and suppression of Wnt activity [231]. KLF4 and KLF5 illustrate another example of opposing functions. KLF4 serves as a negative regulator of canonical WNT signaling by directly binding β-catenin and blocking β-catenin acetylation by p300/CBP, thereby preventing nuclear translocation and transcriptional activity of β-catenin [232,233]. KLF4 is also negatively regulated by miR-92a, which promotes tumorigenesis through activation of Wnt/β-catenin signaling in colorectal cancer [234]. In contrast, KLF5 enhances Wnt activity by interacting with β-catenin and TCF4 to activate Wnt target genes [134,235], and in esophageal squamous epithelial cells, pharmacological inhibition of KLF5 with ML264 promotes β-catenin degradation [78]. Consistent with a WNT-promoting function for KLF5, ablation of *Klf5* in *Lgr5*+ intestinal stem cells of mice reduces expression of WNT target genes [25]. In colorectal cancer, KLF6-positive cells display reduced Wnt activity and diminished stemness, favoring a wound-healing phenotype [236], but in fluorouracil–resistant colorectal cancer cells, KLF6 promotes nuclear β-catenin localization, again highlighting potential context-dependent functions of the KLFs [237]. KLF8 also exhibits dual regulatory capacity, as Wnt3a stimulation induces KLF8 expression, indicating its role as a downstream effector of Wnt/β-catenin signaling, while KLF8 overexpression in HCC cells increases both cytoplasmic and nuclear β-catenin, suggesting an upstream function, and high KLF8 expression together with nuclear β-catenin correlates with aggressive HCC and is associated with enhanced stem-like traits [238,239]. In PDAC, KLF9 suppresses Wnt/β-catenin signaling by downregulating Frizzled-5 [240]. β-catenin acts as a coactivator of KLF11 in gastric cancer, promoting the expression of stemness-related genes [241], and KLF12 activates Wnt/β-catenin signaling by elevating DVL2, thereby enhancing the proliferative and invasive potential of PDAC cells [242]. Together, these findings illustrate the intricate and context-specific roles of KLF family members in modulating Wnt signaling, thereby influencing cell fate, stemness, tumor progression, and therapeutic response.

### 9.4. Notch Signaling

**Figure 7:**
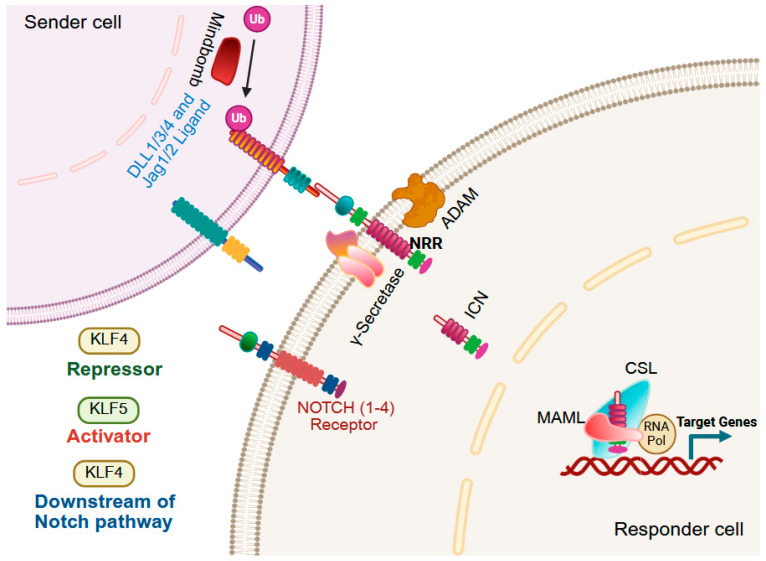
KLFs in the Notch pathway. The Notch pathway, initiated by ligand (DLL1, DLL3, DLL4, Jagged1, Jagged2) binding to Notch receptors (NOTCH1–4), triggers sequential proteolytic cleavages by ADAM metalloproteases (ADAM10 or ADAM17) and γ-secretase, releasing the intracellular Notch domain (ICN). ICN translocate to the nucleus, forming a transcriptional activation complex with CSL and MAML, and recruiting cofactors such as p300 and KDMA1. In the small intestine and colon, Notch1-mediated activation of HES1 represses KLF4, inhibiting goblet cell differentiation. In Barrett’s esophagus, Notch1 inhibition activates KLF4, inducing columnar cell lineage markers and glandular mucins while reducing squamous keratins. Organoid differentiation from colon tumors requires BMP activation to induce enterocyte markers (KLF4, KRT20, CA1, FABP2) and Notch inhibition to promote goblet cell markers (TFF2, TFF3, AGR2). KLF5 supports intestinal stem cell self-renewal and regenerative capacity by regulating Wnt/Notch target gene enhancers; its loss leads to premature differentiation and stem cell depletion. Created with BioRender.com.

The Notch signaling pathway is a highly conserved, contact-dependent pathway that governs cellular differentiation, proliferation, survival, and stem cell maintenance. In mammals, four Notch receptors interact with five canonical ligands, Delta-like (DLL1, DLL3, DLL4) and Jagged (JAG1, JAG2) [243,244]. Ligand binding induces sequential proteolytic cleavages, first by ADAM metalloproteases, followed by γ-secretase-mediated release of the intracellular Notch domain (ICN), which is then translocated to the nucleus to activate Notch-responsive genes. Dysregulated Notch signaling is a hallmark of multiple cancers, where it may act as either a tumor suppressor or an oncogene depending on tissue context [245]. In the gastrointestinal tract, KLFs are important mediators of NOTCH signaling (Figure 7). For example, Notch1-mediated ICN represses KLF4 via the transcriptional repressor HES1, thereby inhibiting goblet cell differentiation, although KLF4 loss or Notch pathway ablation in intestinal stem cells does not impede the differentiation of progenitors into goblet cells in adult mice [140,246,247]. In esophageal epithelial cells, Notch1 inhibition activates KLF4, driving transdifferentiation toward columnar-like cells that express keratins indicative of a Barrett’s esophagus lineage and glandular mucins, while reducing squamous keratin expression [248]. In colon tumor organoid cultures, Notch inhibition favors goblet cell marker expression, while BMP activation promotes enterocyte lineage differentiation [249]. *Klf5* deletion in *Lgr5*^+^ intestinal stem cells reduces NOTCH signaling, and these *Klf5*-null intestinal stem cells fail to produce any secretory lineages [25].

### 9.5. MAPK Signaling

**Figure 8:**
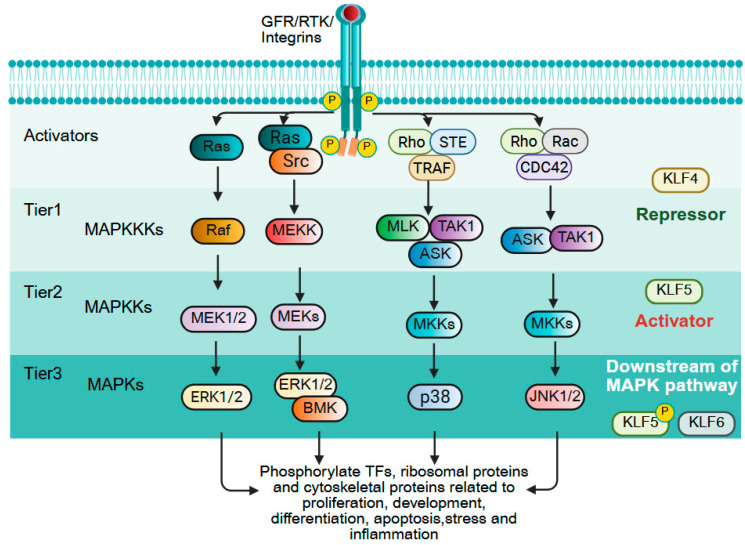
KLFs in MAPK signaling. The mitogen-activated protein kinase (MAPK) cascade is organized into three tiers: MAPKKKs (e.g., Raf, MEKK, MLK, ASK, TAK1), MAPKKs (e.g., MEK1/2, MKK3/6), and terminal MAPKs (e.g., ERK1/2, p38, JNK). Activation occurs through dual phosphorylation of a conserved Thr–X–Tyr motif, transmitting extracellular cues to downstream effectors, including transcription factors and cell cycle regulators. In cancer, hyperactivation of MAPK signaling via mutations (e.g., KRAS, BRAF) or upstream overexpression promotes proliferation, survival, and drug resistance. Krüppel-like factors (KLFs) form bidirectional regulatory loops with MAPK signaling. KLF4 acts as a negative regulator, induced by ERK–E2F1 but suppressing ERK, JNK, and p38 phosphorylation; ERK can also promote KLF4 degradation via ubiquitination. In contrast, KLF5 often amplifies MAPK signaling by transcriptionally activating PDP1 (enhancing BRAF–MEK1 interaction) and EGFR, and by driving cyclin D1 expression downstream of ERK1/2 and p38. Tumor context shapes regulation: in colorectal cancer, PrPC–EGFR–p38 signaling upregulates KLF5 and promotes cisplatin resistance; in KRAS-mutant pancreatic cancer, IL-1β/hypoxia induce KLF5 via p38 independent of ERK. Post-translational modifications further modulate KLF5 activity, with ERK phosphorylation at Ser-406 enhancing stability and p38 phosphorylation at the same site altering transcriptional specificity. MAPK signaling also inactivates KLF6 via splicing regulation, promoting tumor progression. Created with BioRender.com.

The mitogen-activated protein kinase (MAPK) pathway consists of a sequential activation of serine/threonine kinases that governs cell proliferation, differentiation, and survival [250]. The cascade is hierarchically organized into three tiers: (1) MAPK kinase kinases, (2) MAPK kinases, and (3) terminal MAPKs that phosphorylate transcription factors, ribosomal proteins, and cytoskeletal components to drive context-dependent responses. In cancer, MAPK signaling is frequently hyperactivated due to mutations (e.g., KRAS, BRAF) or overexpression of upstream regulators, promoting tumor growth and drug resistance [251]. KLFs both regulate and are regulated by MAPK signaling, creating feedback loops that influence cell fate (Figure 8). KLF4 predominantly functions as a negative regulator of MAPK pathway activation, and ERK-mediated induction of E2F1 promotes KLF4 transcription, leading to elevated KLF4 that suppresses phosphorylation of JNK, ERK, and p38, thereby attenuating MAPK signaling and reducing tumor cell proliferation [252,253]. However, ERK activation can also trigger KLF4 degradation via ubiquitination, indicating a finely tuned regulatory balance [254]. In contrast, KLF5 exerts a context-dependent influence on MAPK signaling, often acting as an amplifier. KLF5 transcriptionally upregulates pyruvate dehydrogenase PDP1, which serves as a scaffold to enhance BRAF–MEK1 interaction, thereby activating MAPK signaling and also induces EGFR expression, further promoting MEK/ERK pathway activity in epithelial cells [251,255]. Downstream, activation of ERK1/2 and p38 drives cyclin D1 expression through KLF5, facilitating G1/S cell cycle progression [256]. MAPK signaling also regulates KLF5 expression in a tumor-specific manner: in CRC, prion protein (PrP^C^) colocalizes with EGFR to activate p38, which upregulates KLF5 and contributes to cisplatin resistance, while *KRAS*-mutated CRC cells exhibit high KLF5 expression via the KRAS–RAF–MEK–ERK axis [257]. Conversely, in KRAS-mutated pancreatic cancer, KLF5 induction is mediated by IL-1β and hypoxia through p38 MAPK signaling, independent of KRAS–ERK activation [258]. Beyond transcriptional regulation, MAPK signaling post-translationally modifies KLF5; for example, ERK phosphorylation at Ser-406 enhances KLF5 transcriptional activity and stability, whereas p38 phosphorylation at the same site diminishes its interaction with retinoic acid receptor (RAR)-α, altering target gene specificity [2,259]. MAPK also influences KLF6 activity, with RAS–MAPK signaling altering its splicing through the SR protein ASF/SF2, leading to inactivation and promoting HCC growth [260,261]. Collectively, the MAPK–KLF regulatory network forms a bidirectional axis in cancer biology, since MAPK activity can reciprocally modulate KLF function through transcriptional control and post-translational modifications. Deciphering these interactions may uncover therapeutic opportunities to jointly target oncogenic MAPK signaling and KLF-mediated transcriptional programs.

### 9.6. TGFβ Signaling

**Figure 9:**
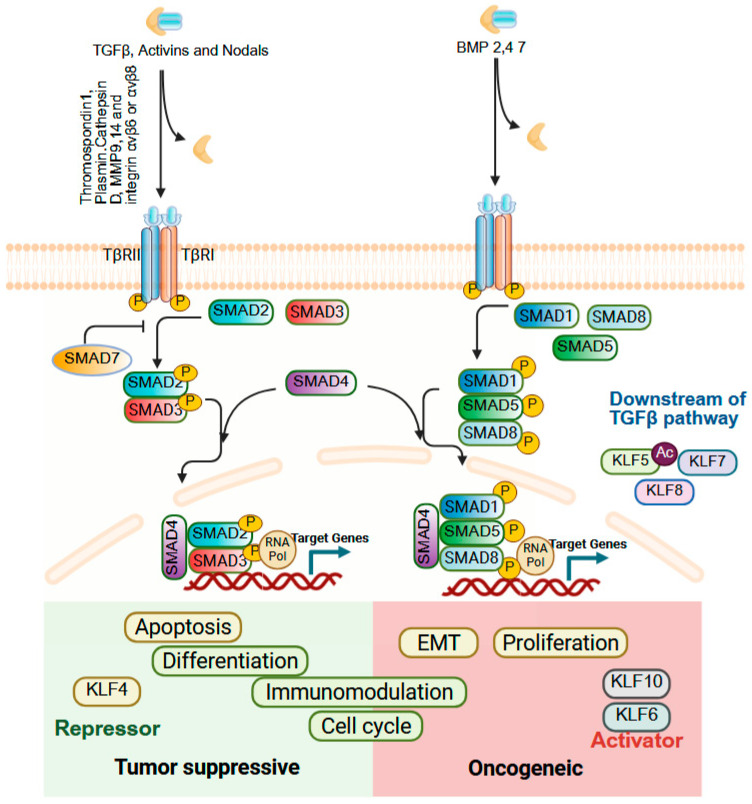
KLFs in TGFβ signaling. The diagram illustrates the dual role of TGFβ signaling in GI tumorigenesis. In the tumor-suppressive phase, active TGFβ or BMP ligands bind type II and type I receptors, phosphorylating SMAD2/3 or SMAD1/5/8, which complex with SMAD4 to induce genes involved in cell cycle arrest and apoptosis. KLF4, a transcriptional target of BMP-2/4/6 and TGFβ1, promotes mesenchymal-to-epithelial transition (MET) by increasing E-cadherin and reducing vimentin and Slug, thereby antagonizing TGFβ-driven epithelial–mesenchymal transition. In the tumor-promoting phase, TGFβ acetylates KLF5 at lysine 369 (AcK369-KLF5), activating CXCR4 and IL-11 secretion via IL-6 signaling, which sustains EMT, enhances BCL2 expression, and confers drug resistance. In colorectal cancer (CRC), mesenchymal stromal cell (MSC)-derived CCL7 activates the KLF5/CXCL5 axis to promote metastasis, while TGFβ suppresses this axis via SMAD4 modulation. KLF6 upregulation of TGFβR1 to enhance EMT, contrasted by platelet-derived TGFβ-mediated KLF6 repression in hepatocellular carcinoma; and KLF8 activation by TGFβ to promote EMT and tumor progression in CRC and gastric cancer. Created with BioRender.com.

Transforming Growth Factor-beta (TGFβ) signaling orchestrates a wide spectrum of cellular processes, including proliferation, differentiation, apoptosis, immune modulation, and tissue homeostasis [262,263]. The TGF-β superfamily comprises three TGFβ isoforms, activins, Nodal, BMPs and several growth and differentiation factors [264,265,266]. These ligands are secreted as latent dimers bound to the extracellular matrix (ECM) and require precise activation mechanisms to be released in their bioactive form, and TGF-β signaling is mediated and regulated by the SMAD family of proteins. In cancer, TGFβ signaling typically displays a dual role: in early stages, it functions as a tumor suppressor by inducing cell cycle arrest and apoptosis, while in advanced stages, it promotes tumor progression by driving EMT, angiogenesis, cancer-associated fibroblast activation, and immune evasion [267]. KLFs are capable of influencing both its tumor-suppressive and tumor-promoting functions of TGFβ signaling (Figure 9). KLF4 and TGFβ exhibit reciprocal regulation, with each able to suppress the other’s activity. KLF4 is a transcriptional target of BMP-2/4/6 and TGFβ1 in vascular smooth muscle cells [268], promoting mesenchymal-to-epithelial transition (MET) by increasing E-cadherin and reducing vimentin and Slug levels thereby counteracting TGFβ-induced EMT [269,270]. Conversely, TGFβ acetylates KLF5 at lysine 369 to activates CXCR4 and drives paracrine IL-11 secretion via IL-6 signaling, which sustains EMT and contributes to drug resistance through BCL2 upregulation [271,272]. In CRC, mesenchymal stromal cell–derived CCL7 enhances KLF5/CXCL5 signaling to promote metastasis, whereas TGFβ suppresses this pathway via SMAD4 modulation [273]. KLF6 facilitates TGFβ-driven EMT by upregulating TGFβR1, yet platelet-derived TGFβ downregulates KLF6 to promote HCC progression [274,275]. KLF8, a transcriptional target of TGFβ, promotes EMT and tumor progression in CRC and gastric cancers [119,161]. Collectively, these findings underscore that TGFβ–KLF interactions form a finely tuned regulatory axis with major implications for cancer biology, fibrosis, and inflammation. The role of other KLF family members in TGFβ signaling within GI cancers remains largely unexplored, and more insights, leading to strategic targeting of this axis could enable therapeutic approaches that preserve the tumor-suppressive actions of TGFβ while minimizing its pro-metastatic effects.

## 10. Current Advancements and Future Perspectives

Recently, there have been significant advances in our understanding of KLFs in gastrointestinal physiology and the development of GI diseases such as GI cancers, driven by both conceptual breakthroughs and emerging technologies. Interestingly, KLFs may function both to drive cellular differentiation and to promote stemness or pluripotency and identifying the mechanisms underlying these distinctive functions of KLFs will be important for the field. In addition, aberrant expression of various KLF family members is a common feature across human malignancies, and specific KLFs have been implicated in nearly every cancer-relevant process, including proliferation, apoptosis, migration and invasion, angiogenesis, metabolic reprogramming, immune modulation, and stemness, highlighting their potential as biomarkers and therapeutic targets. Single-cell approaches and spatial multi-omic techniques now enable high-resolution dissection of KLF expression patterns and functional activity across heterogeneous tumor and normal tissue microenvironments. These approaches should permit mapping of cell type-specific KLF programs, uncover rare KLF-expressing subpopulations, including therapy-resistant or stem-like cells, allow perturbation-aware profiling when integrated with CRISPR or chemical screening platforms, and place KLF activity in anatomical and niche contexts, clarifying how localization (crypt vs. villus, tumor core vs. invasive front) influences downstream signaling and cellular phenotype and elucidating KLF dependencies and synthetic-lethal interactions across diverse genetic backgrounds and microenvironments. Transcription factors had previously been thought to be “undruggable” [276], but several KLFs have already emerged as potential therapeutic targets [277,278], highlighting the possibilities for other KLF family members as well. Traditional strategies such as small-molecule modulators, epigenetic drugs, and antisense oligonucleotides/RNAi remain relevant for indirectly modulating KLF expression or function. More recently, targeted protein degradation technologies such as PROTACs have emerged as promising precision tools capable of eliminating otherwise “undruggable” transcription factors or their essential cofactors, an attractive approach for oncogenic KLFs or pathological KLF complexes [279]. Additional modalities include engineered peptides that disrupt protein–protein interactions, stabilized decoy oligonucleotides that sequester KLF DNA-binding activity, and CRISPR-based gene regulation tools (CRISPRi/CRISPRa) for controlled repression or activation of KLF loci in ex vivo or localized in vivo settings. Moreover, combining KLF targeting with modulation of cooperating pathways (e.g., KLF5 with WNT or KRAS effectors) or integrating KLF inhibition with immune checkpoint therapies of other modulators of the microenvironment may help overcome resistance to single-agent treatments.

Yet, despite these promising developments, several important challenges persist. KLF family members exhibit strong context dependency: the same KLF may act as a tumor suppressor in one tissue or disease stage and as an oncogene in another. This duality complicates therapeutic design and necessitates precise molecular stratification of patients. Functional redundancy and compensatory mechanisms within the KLF family can diminish the efficacy of single-target interventions. Moreover, post-translational modifications (e.g., phosphorylation, acetylation, SUMOylation, ubiquitination) and isoform-specific functions add further complexity, and many of these roles are not yet well defined across various disease settings. In addition, interactions with core tumor regulators (such as p53, KRAS, APC, PTEN, SIN3A, and p21) and chromatin-remodeling complexes often dictate whether a KLF promotes proliferation, differentiation, or apoptosis. Clinical translation of KLF-targeted therapies will require meticulous attention to specificity and safety, given that many KLFs play essential roles in normal tissues, so unsystematic targeting could have significant side effects or even be lethal. Strategies such as spatially restricted delivery, transient modulation, and therapeutic windows that exploit tumor-specific dependencies may be critical. Biomarker-driven patient selection—integrating genomic, transcriptomic, and proteomic data—helps to identify individuals most likely to benefit while avoiding adverse effects in cases where a particular KLF functions as a tumor suppressor.

In conclusion, KLFs occupy nodal positions at the intersection of transcriptional regulation, metabolism, stress response, and cell fate determination in both normal GI physiology and disease, and translational success will depend on the ability to selectively neutralize disease-promoting and oncogenic KLF programs while preserving their essential physiological functions.

## Figures and Tables

**Table 1:** KLF chromosomal location, expression patterns, and interacting proteins.

Gene Symbol	Human Chromosome	Expression in Adult Tissues	Interacting Co-Regulators Based on Experiments and/or STRING Prediction ^#^
KLF1	*chr19:12,884,422-12,887,201*	Erythroid	CREBBP, UBA52, UBB, UBC, RPS27A
KLF2	*chr19:16,324,826-16,328,685*	Lung, blood vessels, lymphocytes	FOXO1, p300, KAT2B, WWP1, FBXW7
KLF3	*chr4:38,664,197-38,701,517*	Adipocytes, brain and erythroid tissue	CTBP2, FHL3, LHX8, UBE2I
KLF4	*chr9:107,484,852-107,490,482*	Gut, skin, cornea, several other epithelial tissues	Sp1, p300, HUWE1, HDAC2, CREBBP
KLF5	*chr13:73,054,976-73,077,541*	Gut, skin, lung, cornea, several other epithelial tissues	RARA, NFkB1, p300, WWP1, FBXW7
KLF6	*chr10:3,775,996-3,785,281*	Ubiquitous	RELA, SP1, TAF9, NFKBIA, HDAC3
KLF7	*chr2:207,074,137-207,173,856*	Ubiquitous	FBXO38
KLF8	*chrX:55,908,123-56,291,531*	Ubiquitous	CTBP1, p300, CREBBP, KAT2B
KLF9	*chr9:70,384,604-70,414,657*	Ubiquitous	Sin3A, PGR
KLF10	*chr8:102,648,784-102,655,725*	Ubiquitous	SIAH1, KAT2B, Sin3A, SP1, FOXP3
KLF11	*chr2:10,042,849-10,054,836*	Ubiquitous	SIN3A, p300, CBX5
KLF12	*chr13:73,686,089-74,306,045*	Bone, brain, kidney, liver and lung	CTBP1, IDO2, DNMT3L, EHMT2
KLF13	*chr15:31,326,835-31,435,665*	Ubiquitous	SIN3A, HDAC1, CREBBP, KAT2B, MMP28
KLF14	*chr7:130,415,525-130,418,967*	Ubiquitous	SP1, PAX3, GATA1, Pou3F1,RXRB, ZEB1
KLF15	*chr3:126,288,125-126,357,408*	Ubiquitous	STAT3, p300, ANKS1A, ZBTB24, PRKAB2
KLF16	*chr19:1,852,399-1,876,536*	Ubiquitous	BPGM, H4C6, p300, SIN3A and Sin3B
KLF17	*chr1:44,043,927-44,135,140*	Ubiquitous	CIB3
KLF18	*chr1: 44,137,821-44,141,631*	Testis, upper leg skin	AQP1, ATG, BTN2A, BTN2A2, CCM2

# Based on string software.

**Table 2:** KLFs in gastrointestinal cancers.

Gene Symbol	Role	Cancer Types	Main Mechanism
KLF1	Oncogene	Gastric, CRC	Drives proliferation
KLF2	Tumor suppressor	CRC, PDAC	Regulates angiogenesis and HIF-1α, Notch-1, GPX4 pathways
KLF3	Tumor suppressor	Gastric, ESCC, Pancreatic	Transcriptional repressor; KLF3-AS1 (lncRNA) suppresses tumors
KLF4	Dual* (mainly tumor suppressor in GI)	Gastric, CRC, PDAC	Regulates p21/p27/p53; inhibits EMT; suppresses proliferation and invasion
KLF5	Dual* (oncogene/tumor suppressor)	ESCC, CRC, Gastric	Oncogenic TP63/SOX2 complex; drives metabolism; suppresses ferroptosis
KLF6	Dual* (oncogene/tumor suppressor)	Gastric, Intestinal, HCC	Tumor-suppressive via p21; silenced by the TAM–UHRF1 axis in HCC
KLF7	Oncogene	CRC, HCC, Gastric	Activates PDGFB leading to MAPK/ERK, PI3K/AKT, JAK/STAT3; promotes invasion
KLF8	Oncogene	Gastric, CRC, PDAC	Induced by TGFβ1/hypoxia; promotes EMT, invasion, drug resistance
KLF9	Tumor suppressor	ESCC, Gastric, CRC	Represses β-catenin/TCF and MMP28; reduces metastasis and drug resistance
KLF10	Tumor suppressor	Gastric, ESCC	Effector of TGFβ/SMAD; represses SLUG; loss leads to poor prognosis and radioresistance
KLF11	Oncogene	Gastric	Promotes invasion via Twist1
KLF12	Dual* (oncogene/tumor suppressor)	ESCC, Gastric, Rectal	Oncogenic in gastric; suppressor in rectal (regulates L1CAM, metastasis)
KLF13	Oncogene	Esophageal, Gastric	Promotes EMT and invasion via NF-κB and GPIHBP1
KLF14	Tumor suppressor	CRC	Represses glycolysis (LDHB) and centrosome amplification (Plk4)
KLF15	Tumor suppressor	Gastric, CRC	Regulates p21/p57; lncRNAs (TFAP2A-AS1, LINC00689) suppress YAP1/β-catenin
KLF16	Oncogene	CRC	Supports stress tolerance via ATF4 translational reprogramming
KLF17	Tumor suppressor	CRC, Gastric	Activates FHL1; inhibits EMT and chemoresistance; silenced via hypermethylation

* The identified functions in GI cancer depend on context such as organ or tissue type.

## Data Availability

Not applicable.
